# Measuring 3D Hand and Finger Kinematics—A Comparison between Inertial Sensing and an Opto-Electronic Marker System

**DOI:** 10.1371/journal.pone.0164889

**Published:** 2016-11-03

**Authors:** Josien C. van den Noort, Henk G. Kortier, Nathalie van Beek, DirkJan H. E. J. Veeger, Peter H. Veltink

**Affiliations:** 1 Biomedical Signals and Systems Group, MIRA Research Institute for Biomedical Technology and Technical Medicine, University of Twente, Enschede, the Netherlands; 2 Department of Human Movement Sciences, Faculty of Behaviour and Movement Sciences, Vrije Universiteit Amsterdam, MOVE Research Institute Amsterdam, Amsterdam, the Netherlands; University of Chicago, UNITED STATES

## Abstract

Objective analysis of hand and finger kinematics is important to increase understanding of hand function and to quantify motor symptoms for clinical diagnosis. The aim of this paper is to compare a new 3D measurement system containing multiple miniature inertial sensors (PowerGlove) with an opto-electronic marker system during specific finger tasks in three healthy subjects. Various finger movements tasks were performed: flexion, fast flexion, tapping, hand open/closing, ab/adduction and circular pointing. 3D joint angles of the index finger joints and position of the thumb and index were compared between systems. Median root mean square differences of the main joint angles of interest ranged between 3.3 and 8.4deg. Largest differences were found in fast and circular pointing tasks, mainly in range of motion. Smallest differences for all 3D joint angles were observed in the flexion tasks. For fast finger tapping, the thumb/index amplitude showed a median difference of 15.8mm. Differences could be explained by skin movement artifacts caused by relative marker movements of the marker system, particularly during fast tasks; large movement accelerations and angular velocities which exceeded the range of the inertial sensors; and by differences in segment calibrations between systems. The PowerGlove is a system that can be of value to measure 3D hand and finger kinematics and positions in an ambulatory setting. The reported differences need to be taken into account when applying the system in studies understanding the hand function and quantifying hand motor symptoms in clinical practice.

## Introduction

The human hand is important in many daily life activities and fine motor tasks. Performance of these tasks can become compromised with age and disease.

With aging, the biomechanical function and neurophysiological characteristics of the extrinsic hand muscles, which are responsible for individual finger coordination, may change and result in less finger-independency and a change in muscle interactions (passive coupling between muscle structures and neural control) [1–3]. To further increase our understanding of these phenomena, studying the finger-interdependency during various finger movement tasks in healthy young and aged subjects is important [4].

In clinical practice, assessment of hand motor symptoms and performance of hand motor tasks are of relevance in diseases such as Parkinson’s Disease (PD) to assess the neurological state of a patient [5], rheumatoid arthritis of the hand [6], arthritis of the carpometacarpal joint (in the thumb) [7] or evaluation of surgery in e.g. tendon transfer [8]. Clinicians often use clinical rating scores. However, assessment is highly dependent on experience which makes it hard to interpret the outcomes [9,10]. An objective and reliable quantification of the hand motor symptoms could improve the clinical scoring to support clinical decision making and objective evaluation of treatment.

Several systems have been developed to measure hand and finger kinematics, such as camera-based marker systems and instrumented gloves (e.g. piezo-resistive bend sensors or optical fiber sensors) [11]. The disadvantages of most of these systems are limited accuracy, need for complex calibration, line of sight problems (opto-electronic markers), crosstalk due to misalignment of sensors, poor robustness, or limited usability during functional tasks or in clinical practice [12–14].

Recently, a measurement system to assess 3D hand and finger kinematics based on multiple miniature inertial and magnetic sensors has been proposed: the PowerGlove [12]. It enables a 3D reconstruction of all finger joints and orientation of the hand by using an extended Kalman filter algorithm that fuses all sensory inputs and a biomechanical hand model. The PowerGlove might enable an accurate measurement of finger dynamics and objective quantification of hand motor symptoms, which are of interest in elderly and patient populations.

To apply the PowerGlove for quantitative functional evaluation of hand and finger coordination, a validation for specific movement tasks is required. Prior evaluations of accuracy have been limited to the index and thumb fingertip-positions during flexion, circular and pinch motions (root mean square (RMS) difference with opto-electronic markers: 5.0–12.4mm), and to repeatability tests for total joint angles in flat and flexed hand position (range difference: 0.7–1.9deg), in which also a comparison in outcome measures with other data gloves has been made [12]. However, evaluation of the performance of the PowerGlove in a wider range of movements, especially focused on the 3D joint angles of the fingers, is necessary.

The aim of this study is therefore to evaluate the PowerGlove for the accurate measurement of various finger motor tasks in terms of flexion of the metacarpophalangeal (MCP), proximal and distal interphalangeal (PIP and DIP) joints of the index finger and position of the tip of the thumb and index finger. Additionally, index finger ab/adduction movements and circular pointing movements are included to evaluate measurement out of the sagittal plane, i.e. ad/adduction and rotation angles. 3D joint angles and fingertip positions measured with the PowerGlove are compared to kinematic data measured simultaneously with an opto-electronic marker system, a system that is often used for quantification of movement.

## Methods

### Subjects

Three healthy subjects participated (age 29.3±3.1y, BMI 25.4±7.4kg/m^2^). The experiment was performed at the movement laboratory of the department of Human Movement Science of the VU University Amsterdam, Netherlands. The ethical board of the faculty approved the study protocol. The medical ethical committee of the Medisch Spectrum Twente (Enschede, NL) confirmed that no further ethical approval concerning the Medical Research Involving Human Subjects Act (WMO) was required, due to the nature of the study (technical). Written informed consent was obtained from all participant included in the study.

### PowerGlove

**Fig 1:**
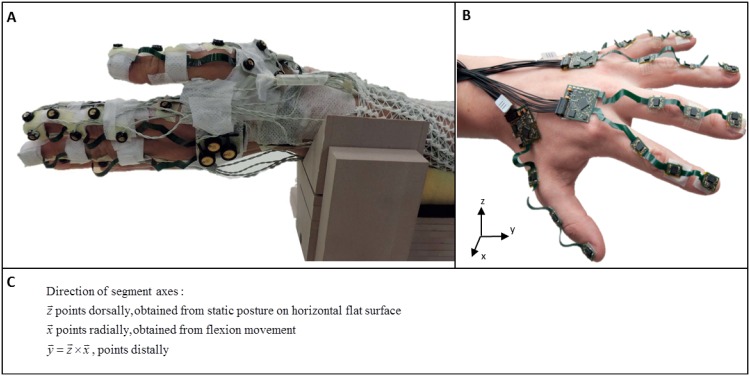
Set-up of the validation experiment: 23 opto-electronic markers (Optotrak). (A) and 11 inertial sensor units of the PowerGove [12] (B) were placed on the hand and finger segments of the thumb, index and middle finger. Sensor units on the ring and small fingers were not applied in this study. The arm was placed on a custom-made arm-rest with a palmar position of the hand of 45deg. The directions of the segment axes are described in (C).

Eleven inertial sensor units, each containing a 3D gyroscope and a 3D accelerometer (ST LSM330DLC) [12] were attached to the dorsal side of the left hand (2 sensors), on the metacarpal, proximal and distal phalanges of the thumb (3 sensors) and the proximal, middle and distal phalanges of the index (3 sensors) and middle fingers (3 sensors) using small Velcro straps (Fig 1B).

An anatomical calibration procedure was performed to determine the sensor-to-segment coordinate systems of the PowerGlove. This calibration procedure included several steps. First, the hand was placed on a flat horizontal surface (i.e. a table) for a few seconds, in such a way that the gravity vector was perpendicular to the palm of the hand, the fingers in neutral position with the phalanges aligned to each other, and the thumb in abduction (without overstretching [15]). Secondly, the thumb was placed on the table for a few seconds with the dorsal side of the thumb and the nail positioned horizontally, in such a way that the gravity direction was perpendicular to the long axis of the thumb. Then, the thumb was flexed three times in the IP joint (interphalangeal), followed by three flexions of the finger MCP joints (fingers stretched, hand still), and three flexions of all finger joints (MCP, PIP and DIP; hand still). In between each movement the hand was placed on the table. The z-axes of the hand and finger segments were determined from the static postures of hand and thumb, pointing dorsally. The x-axes were determined from the flexion/extension movements and pointed radially. The y-axis followed from the cross-product of z and x and pointed distally (Fig 1C). Finally, the hands were placed together and moved in an eight-shaped movement for five seconds. Angular velocity measured on various hand segments were assumed equal during this movement but measured in different coordinate frames. Relative orientation was deduced, to express the signals of both units on the hand (thumb and index/middle) in a common reference frame [12].

To obtain joint orientations and segment positions from the actual movement trials, data of the PowerGlove were processed in an algorithm (custom-made, MATLAB-based) applying the anatomical segment calibration described above and information from gyroscopes, accelerometers and magnetometers from the movement trials. Primary inputs were angular velocities obtained from the gyroscopes, which were integrated to relative orientations and continuously corrected using biomechanical information (joint constraints in which the joints have limited degrees of freedom), common accelerations and angular velocities, and inclination information obtained from accelerometer. To determine the positions and orientation of various joints and fingertips forward kinematics were applied using length of the finger segments of the subjects as additional input. These segment lengths were measured using measurement tape (long fingers: mid of the MCP joint to the PIP joint, PIP to DIP joint, DIP joint to the tip of the finger; thumb: mid of the CMC joint (carpometacarpal) to the MCP joint, MCP to IP joint, IP joint to tip of the finger). [12]

### Opto-electronic system

Twenty-three active markers of an opto-electronic system (Optotrak 3020, Northern Digital Instruments, Canada) were attached to the lower arm (data not used in this study); hand and metacarpal of the thumb (clusters of three markers); and to the proximal, medial and distal phalanges of the index and middle fingers and proximal and distal phalanges of the thumb (Fig 1A). For the index and middle finger, markers were placed on the dorsal-radial side of the joints (MCP, PIP, DIP and tip of the finger) aligned in longitudinal finger direction. For the index finger one additional marker was placed on top of the inertial sensor unit resulting in three markers per segment. For the thumb, besides the cluster on the metacarpal, markers were placed on the MCP and IP joint and on the fingertip.

Small finger segment size and limited space due to the placement of the inertial unit caused the use of three separate markers per segment instead of one rigid cluster with three markers on the phalanges.

To estimate segment coordinate systems for the hand and fingers a few calibration steps were taken comparable to the calibration procedure of the PowerGlove: static hand posture with forearm (mid-pronation) and hand on the table, and a flexion/extension movement in all finger joints. From the static posture, the vertical z-axis was determined for all segments, pointing dorsally. From the flexion movement, the instantaneous helical axis [15,16] was calculated for each joint (x-axis, pointing radially) (Fig 1C). In this way, anatomical coordinate systems of the opto-electronic marker system were closest to anatomical coordinate systems of the PowerGlove, however not measured simultaneously.

### Protocol

Subjects were asked to perform the finger movements as listed in Table 1. During the flexion tasks, the arm was placed on a custom-made arm-rest with a palmar position of the hand of 45deg for a correct inclination angle necessary for the hand accelerometer unit (Fig 1A). Data were recorded with a sample frequency of 100Hz.

**Table 1:** Finger movement tasks.

Flexion tasks (0.5±0.1Hz; max 0.9Hz)	Fast tasks (1.3±0.9Hz; max 4.2Hz)
(1) MCP flexion of the index finger	(10) MCP/PIP/DIP fast flexion of the index finger
(2) PIP/DIP flexion of the index finger	(11) finger tapping with index and thumb without wrist flexion
(3) MCP/PIP/DIP flexion of the index finger	(12) finger tapping with index and thumb with wrist flexion movements
(4) MCP flexion of the middle finger	(13) hand open/close movements
(5) PIP/DIP flexion of the middle finger	
(6) MCP/PIP/DIP flexion of the middle finger	**Ab/adduction task (0.5±0.0Hz; max 0.5Hz)**
(7) MCP flexion of the index and middle finger	(14) MCP ab/adduction of the index finger
(8) PIP/DIP flexion of the index finger	
(9) MCP/PIP/DIP flexion of the index finger	**Circular pointing task (1.6±0.2Hz; max 1.8Hz)**
	(15) circular pointing of the index finger (movement in MCP joint)

MCP: metacarpophalangeal joint; PIP: proximal interphalangeal joint; DIP: distal interphalangeal joint

Similar tasks (1–10) are of importance in the study of finger interdependency in the aging population [3,4,17]. Tasks 11–13 are examples from clinical tests in diseases such as PD [5]. Finger ab/adduction and circular pointing (tasks 14 and 15) were included since accurate measurement of ab/adduction and rotation is also important for application of the PowerGlove (i.e. measurement of 3D joint angles).

Most tasks (1–9 and 14) were performed at a movement frequency of 0.5Hz using a metronome. Fast flexion, tapping and hand open/close tasks were performed as fast as possible. Movement velocity of pointing was not instructed. Each task was performed 3 times (i.e. 3 trials), with 10 repetitions within a trial for tasks 1–9, 14 and 15. Tapping and hand open/close tasks were performed for a duration of 30 seconds.

### Data analysis

To compare the PowerGlove with the opto-electronic system, 3D angles of the MCP, PIP and DIP joints of the index finger measured with both systems during all tasks as listed in Table 1 were analysed. In addition, the position of the tip of the thumb with respect to the tip of the index finger (i.e. an amplitude) was analysed for the finger tapping tasks (tasks 11 and 12). Data of the middle finger could not be analysed due to limited visibility of the markers, but the small movements of the index finger during middle finger flexion were included in the analyses. Rotation matrices describing the relative joint orientations were decomposed in 3D joint angles following the Grood & Suntay sequence (flexion/extension; ab/adduction; exo/endorotation).

Posthoc cross correlation of joint angles and fingertip positions was used to synchronize data of the PowerGlove with data of the opto-electronic markers. For each joint angle of each task, the root mean square difference (dRMS), offset (mean difference), RMS minus offset (dRMS-offset) and range of motion difference (dROM) were calculated between the two systems over the MCP, PIP and DIP joint angles. The same outcome parameters were calculated for the amplitude between the thumb and index finger tips.

To explain possible differences between the both systems, additional analyses were performed by evaluation of internal consistency of movement reconstruction. First, relative marker movement of the three opto-electronic markers with respect to each other on a single finger segment were calculated (distance in mm). These three markers were assumed to represent a rigid body, although not rigidly connected by a cluster due to the limited space on and small size of each finger segment. However, skin movement artefacts or noisy spatial measures due to use of multiple cameras or marker occlusion may have caused slight movements of the markers with respect to each other [18,19]. This could have affected the coordination system of the rigid body, especially when the markers were placed on small segments. In this study, the markers were placed with a maximal distance of 45mm (proximal phalanx), 24mm (medial phalanx), and 20mm (distal phalanx). To analyse the effect of marker movement on the segment coordinate systems of those phalanges, a method based on singular value decomposition (svd, described in [19]) was applied. The relative movement of the markers on the segment was calculated with respect to its starting position (i.e. hand flat), assuming no movement of markers with respect to each other during a movement task (i.e. assuming a rigid body). The new rotation matrix derived from the newly determined marker movements was then compared to the actual rotation matrix derived from the actual marker movements by calculating the angles between the individual axes that were determined by the markers (smallest angle between the vectors), as described in [20] and [15]. The mean marker movements and their effect on segment orientation were calculated for all trials and all index finger tasks, averaged over the three finger segments in all planes.

Second, to quantify errors in the processing algorithm of the PowerGlove [12], angular velocity measured directly by the gyroscope (expressed in segment axes) was compared to angular velocity derived from the relative segment orientation after sensor fusion filters were applied. The dRMS of the norm of 3D angular velocity was calculated with respect to maximal angular velocity measured for all trials and all tasks, averaged over the three finger segments.

## Results

**Fig 2:**
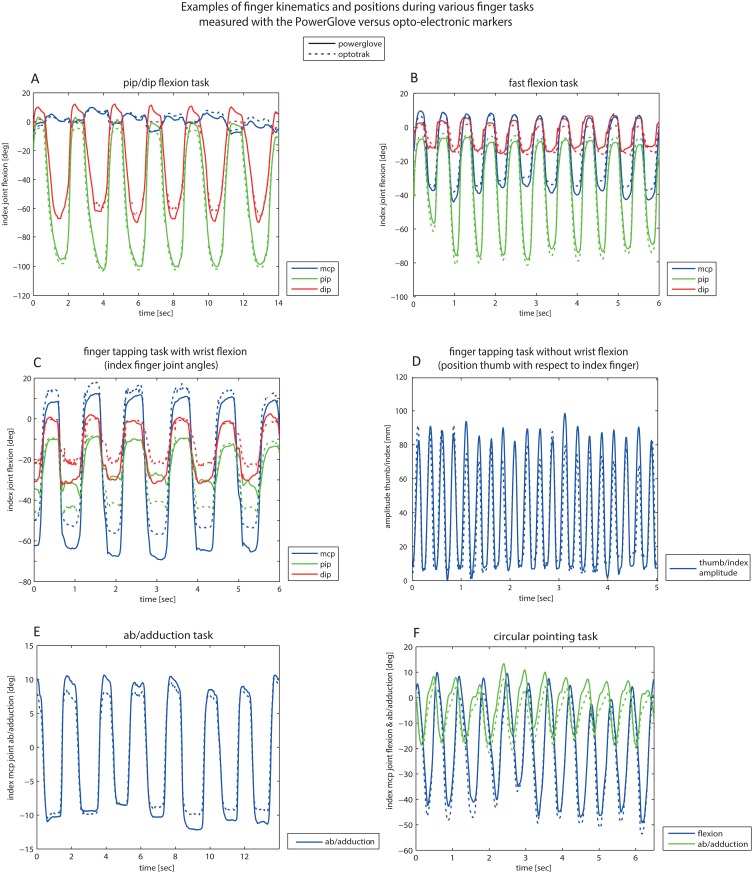
Examples of finger kinematics and positions during various finger tasks, measured simultaneously with the PowerGlove (solid lines) and opto-electronic markers (Optotrak system, dashed lines). (A) index PIP/DIP flexion task with the largest joint flexion in the PIP joint; (B) fast task: index MCP/PIP/DIP flexion task with the largest joint flexion in the PIP joint; (C) thumb/index tapping task with wrist flexion, showing the index finger joint angles with the largest joint flexion in the MCP joint; (D) thumb/index tapping task without wrist flexion, showing the amplitude between the tip of the thumb and index finger; (E) index mcp ab/adduction task, showing the MCP joint ab/adduction angle; and (F) index circular pointing task showing both MCP joint flexion and ab/adduction angles.

Examples from data of the PowerGlove and the opto-electronic marker system during the various tasks are shown in Fig 2.

**Fig 3:**
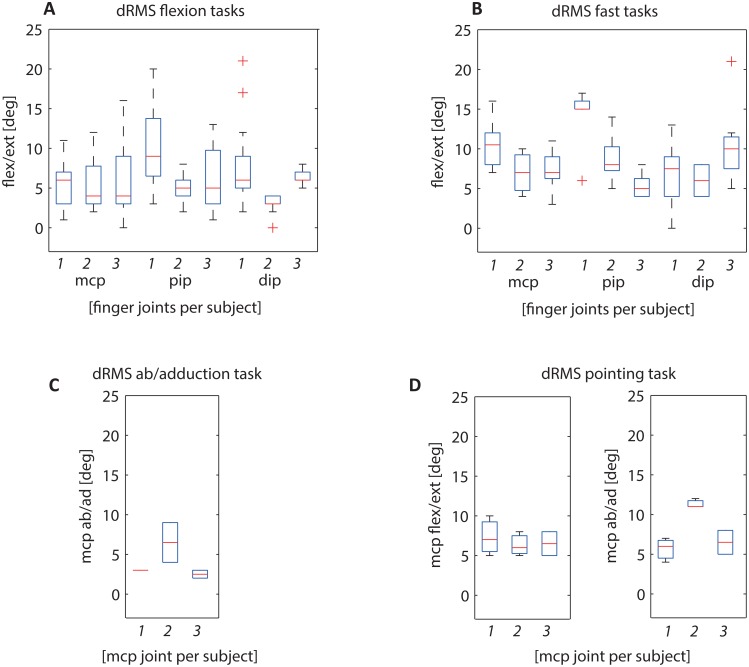
Boxplots of root mean square differences (dRMS) for index finger kinematics (mcp/pip/dip joint angles) between the PowerGlove and the opto-electronic marker system of all 3 included subjects separately per task (1,2,3). Flexion task boxplot includes 15–23 trials per subjects (A), fast task boxplot includes 5–9 trials per subject (B), ab/adduction task boxplot includes 1–2 trials per subject (C) and circular pointing task boxplot includes 2–3 trials per subject (D).

The median dRMS of the main angles of interest of the index finger ranged between 3.3 and 8.4deg over all subjects and tasks (Table 2 and Fig 3; some movement trials had to be excluded from analysis due to marker occlusion) (see also S1 File).

**Table 2:** Outcomes (median and standard deviation) of the comparison of the PowerGlove versus the opto-electronic marker system for the index finger during various finger kinematic tasks and for the thumb tip position with respect to the index fingertip position.

	flexion tasks *(54 trials)*			fast tasks *(20 trials)*			ab/adduction task *(5 trials)*	circular pointing task *(8 trials)*		thumb/index finger tapping task *(7 trials)*
	MCP [deg]	PIP [deg]	DIP [deg]	MCP [deg]	PIP [deg]	DIP [deg]	MCP [deg]	MCP [deg]		Amplitude [mm]
**main angles of interest**	**flexion/extension**			**flexion/extension**			**ab/adduction**	**flexion/extension**	**ab/adduction**	
*dRMS*	5.0 (3.3)	7.3 (4.3)	5.6 (3.7)	8.4 (3.2)	7.1 (4.7)	7.7 (4.2)	3.3 (2.8)	6.6 (1.9)	7.5 (2.9)	15.8 (9.2)
*dRMS-offset*	3.2 (3.1)	4.5 (3.2)	2.6 (2.0)	5.1 (2.6)	7.2 (3.4)	6.1 (1.5)	2.6 (0.8)	6.0 (1.8)	5.3 (2.0)	15.6 (4.5)
*offset*	-1.1 (6.2)	-3.1 (4.1)	-5.1 (4.9)	0.9 (6.7)	-0.2 (7.6)	-2.0 (7.7)	2.2 (4.2)	1.0 (3.3)	-5.4 (5.6)	-2.8 (12.2)
*dROM*	6.6 (5.6)	-4.6 (6.4)	6.6 (5.7)	8.0 (6.9)	-8.7 (6.2)	11.6 (7.3)	2.7 (1.4)	7.6 (7.4)	6.1 (3.0)	10.4 (27.7)
*ROM OT*	46.0 (21.4)	62.5 (30.7)	26.0 (21.0)	71.8 (15.2)	80.2 (20.4)	43.6 (19.3)	24.7 (2.8)	61.1 (12.4)	27.6 (3.3)	108.8 (43.5)
**other angles**										
	**ab/adduction**			**ab/adduction**			**flexion/extension**			
*dRMS*	3.8 (1.5)	1.7 (0.9)	2.7 (1.6)	4.0 (2.5)	3.1 (1.0)	3.3 (2.3)	3.7 (2.6)			
	**exo/endorotation**			**exo/endorotation**			**exo/endorotation**	**exo/endorotation**		
*dRMS*	2.9 (2.2)	1.6 (1.1)	1.3 (1.1)	4.3 (2.4)	3.2 (1.7)	2.1 (2.9)	10.6 (2.6)	4.8 (2.7)		

Values are expressed as median (standard deviation) in degrees of the joint angles (mcp, pip and dip finger joints) and in mm of the thumb/index position

MCP: metacarpophalangeal joint; PIP: proximal interphalangeal joint; DIP: distal interphalangeal joint

dRMS = difference in root mean square

dRMS-offset = difference in root mean square minus offset

offset = mean difference over the curve

dROM = difference in range of motion

ROM OT = range of motion as measured with opto-electronic marker system

Largest differences in the main angles of interest were found in the fast tasks and in the circular pointing task. Smallest differences in the main angles of interest were found in the MCP ab/adduction angles of the ab/adduction task (3.3deg, which is 13% with respect to the total range of motion (ROM) of 24.7deg), however this task showed high differences in exo/endorotation angles (10.6deg). The PIP flexion angle during the fast task showed the smallest difference with respect to the total ROM, i.e. 9% (7.7deg dRMS and 80.2deg ROM OT). On average, the smallest differences in 3D joint angles were found in the flexion tasks (1.3–7.3deg), particularly in subject 2 (Fig 3).

Differences in RMS of the joint angles were mainly caused by a difference in range of motion (dROM), particularly in the fast tasks. An offset (i.e. a mean difference) >5deg was observed for the DIP flexion angle of the flexion tasks and for the MCP ab/adduction angle of the circular pointing task (with a small ROM of 26deg this results in a difference of 21% max ROM).

The thumb tip position showed a dRMS of 15.8mm, also mainly caused by a difference in ROM (Table 2, dROM 10.4mm).

**Fig 4:**
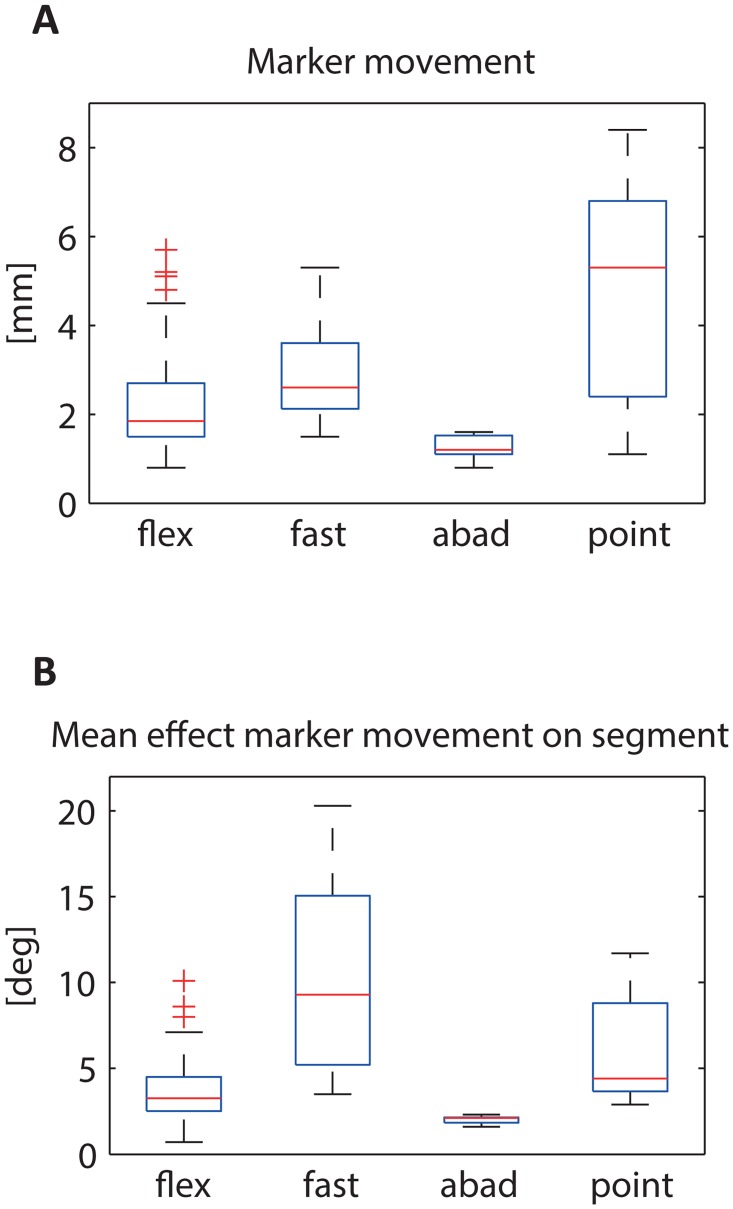
Boxplots of relative marker movement of the 3 markers on a finger segment (averaged over proximal, medial and distal phalanges of the index). (A); and the mean effect on the segment orientation measured with the opto-electronic marker system (averaged over the 3 phalanges) (B) (flex = index finger flexion tasks; fast = fast flexion tasks; abad = ab/adduction task; point = circular pointing task).

Mean marker movement between the opto-electronic markers on a segment was largest for the circular pointing task (1.1–8.4mm) and the fast tasks (1.5–5.3mm) (Fig 4). This also resulted in the largest effect on segment orientation and joint angles (Fig 3) for these two tasks, particularly for the fast tasks (about 10deg) in subject 1 (see also S2 File).

**Fig 5:**
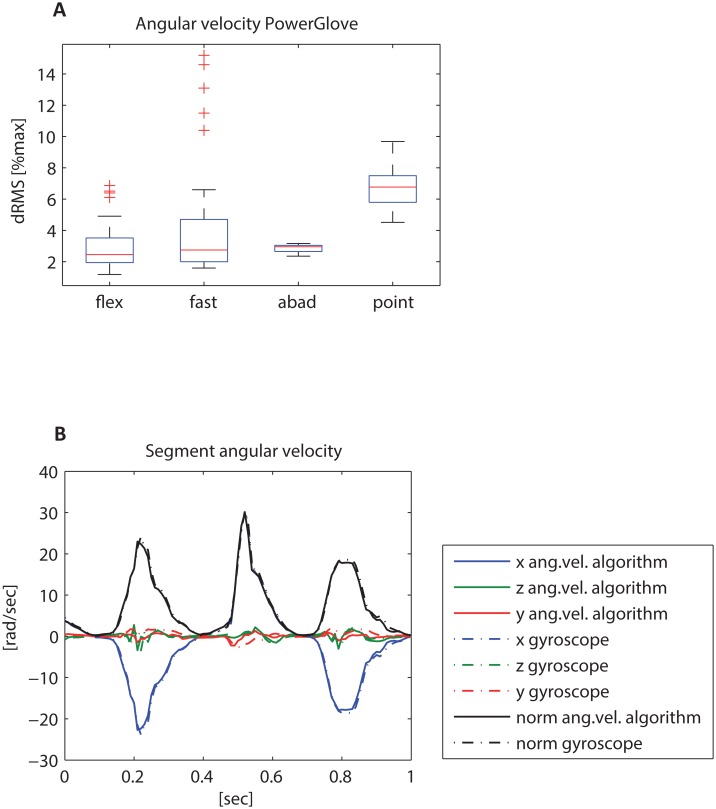
Root mean square difference (dRMS). (A) of the norm of angular velocity as measured directly with the gyroscopes of the PowerGlove versus angular velocity determined from the segment orientation derived from the PowerGlove algorithm after processing of the data (expressed as percentage of maximal angular velocity) for the different movement tasks (flex = flexion tasks; fast = fast flexion tasks; abad = ab/adduction task; point = circular pointing task). (B): example of the different signals during a fast flexion task showing good correspondence.

Angular velocity measured directly by the gyroscopes of the PowerGlove showed a median dRMS below 7%max compared to the angular velocity derived from the segment orientations for all tasks (Fig 5). Largest median difference was found in the pointing task (median 6.8%max), whereas also the fast task showed some individual differences >10%max (see also S3 File).

## Discussion

This paper compares inertial sensing (the PowerGlove) as an instrument for measurement of various finger movement tasks to an opto-electronic system. Tasks like these are relevant for the assessment of finger-interdependency in elderly and for assessment of hand motor symptoms in clinical practice. On average dRMS between the PowerGlove and an opto-electronic marker system used as reference system (Optotrak) ranged between 3 and 8deg. Larger differences observed in fast tasks such as tapping and in circular pointing tasks were mainly expressed in differences in measured range of motion. Therefore, finger kinematics measured by inertial sensing is most comparable to an opto-electronic system during performance of slow flexion tasks.

To explain the differences, the internal consistency of movement reconstruction of both the opto-electronic marker system and the PowerGlove has been evaluated. First, an important factor that explains differences between the systems is high relative marker movement (up to 8.4mm) of markers of the opto-electronic system on one segment that is assumed to be rigid, causing inaccurate segment orientation measured by the system which is used as a reference [15,18]. This indicates that the opto-electronic system has its own limitations. Particularly in fast tasks, skin movement artefacts caused marker movements largely influencing the segment orientation (about 10deg), which resulted in inaccurate measured joint angles by the opto-electronic system. Therefore, differences between the PowerGlove and the opto-electronic system cannot be explained by inaccuracy of the PowerGlove only. Optimal validation of a movement analysis system would only be possible by measuring the movements of the bones itself, e.g. using fluoroscopy [21]. However, the opto-electronic system is currently used in many movement laboratories worldwide and a comparison against such a system provides most insight in the performance of the PowerGlove.

Second, by evaluating the internal consistency of movement reconstruction of the PowerGlove algorithm [12], using a comparison of the angular velocity directly measured with the gyroscope and angular velocity obtained from the segment orientation after processing, highest inconsistency was found for the pointing tasks and fast tasks. Therefore, the PowerGlove also seems to be less accurate for these tasks, although differences were relatively small. Similar to the opto-electronic marker system, skin movement artefacts might have been present and affecting the orientation of the inertial sensor units. Besides, fast movement may lead to clipping of measured signals of the gyroscopes (i.e. reaching its maximum of 2000 deg/s), which will result in differences in range of motion and offsets. This is mainly seen in DIP flexion angles. Furthermore, in fast movement tasks with large relative angular velocity differences (rapid flexion/extension or tapping), the algorithm does not benefit from any filter correction steps, i.e. estimated orientation does almost completely rely on integration of the gyroscope signals. Integration is started from an estimated initial which can be deteriorated as the static orientation might be partly unknown. Moreover, inclination information from the hand accelerometer, used to update the orientation, could not always be used since the hand was not continuously in a correct inclination angle (i.e. at least 45deg as supposed using the arm-hand rest (Fig 1)). In circular pointing and ab/adduction, use of joint constraints (biomechanical model imposed by hand morphology [12] including limited degrees of freedom) still allowed measurement of ab/adduction in the MCP joint. Depending on the hand’s acceleration, angular velocity and inclination angles of different finger segments, parts of the ab/adduction and flexion/extension angles could be updated.

Third, joint angle estimation is dependent on the anatomical segment calibration. Although the same approach was used for both systems, i.e. use of the helical axis method, a static reference posture and comparable segment axes definitions, data for the anatomical calibrations of the two systems were not simultaneously recorded (due to problems with marker visibility during the PowerGlove calibration). Therefore, differences could have occurred at joint axes orientation. Measurement of finger kinematics by different calibration methods and different systems has been studied in several other papers. However, although inertial sensors have been used in different applications like clinical gait analysis [22] or upper extremity movements [20,23], comparisons of performance of inertial sensors with respect to opto-electronic markers for measurement of finger kinematics have not been studied previously. A few studies used opto-electronic markers systems to evaluate anatomical calibrations for finger kinematics. Coupier et al. [16] showed a deviation up to 4deg in joint angles between examiners when using the helical axis method, with a higher reproducibility for the MCP and PIP joints than for the DIP joint. Another study showed that different definitions of anatomical coordinate systems (helical axis/functional movement, reference posture and bony landmark based) resulted in total deviations between different segment axes between 14 and 22deg [15], comparable to the effect we found of relative marker movement in the fast task (Fig 4). The choice of anatomical coordinate system highly affected the ab/adduction and rotation angles of the joints during flexion tasks (mean differences 4-11deg) [15]. Although we have been using the same definitions of anatomical coordinate systems for both systems, part of the differences in joint angles between systems could be explained by the performance of the segment calibration. For example, the difference in ab/adduction angles during circular pointing tasks in subject 2 (Fig 3), could not only be explained by relative marker movement but might have been caused by the segment calibration. A third study used a minimal set of markers (only on hand and distal phalanges of thumb and index) in combination with a mathematical model, to compare MCP, PIP and DIP angles with data obtained from a complete set of markers on all the individual segments [13]. The mean differences they found in joint angles between the two methods (8-17deg) exceeded the differences we found between PowerGlove and opto-electronic system.

In addition to joint angles of the index finger, the position of the tip of the thumb with respect to the tip of the index finger was evaluated, expressed as an amplitude, showing a dRMS of 15.8mm. Previously, a dRMS of 5mm and 12.4mm for index finger tip position was reported in respectively flexion and circular pointing tasks [12]. The somewhat larger difference observed for the thumb/index amplitude in our study might be caused by involvement of two fingers with their own position errors summing up. Any differences between the two systems in thumb positions and index positions can be related to the measurement accuracy of the finger segment lengths using measurement tape, needed for the forward kinematics in the data processing of the PowerGlove; biomechanical modelling of the thumb using inertial sensors based on orientation of the CMC joint in combination with the anatomical segment calibration; the fast movement of the thumb and index during tapping reaching the maximum of the range of the gyroscopes; or accuracy of opto-electronic marker position measurement and skin movement artefacts. Due to the many degrees of freedom and the skin tissue movement in and around the CMC and MCP joints of the thumb, this fingers remains the most difficult to measure using systems that are based on orientation only, like inertial sensor. Whether the reported difference is consider to be large or not depends on the application and measure of interest.

### Application

The PowerGlove is a system that can be of value for measurement of various finger movement tasks in a variety of conditions and population. In finger movement tasks relevant for the study of finger-interdependency in elderly, small movements that occur in the non-instructed finger(s) are of great interest in addition to large movements in instructed finger(s) [3]. Small and slow flexion movements can be measured accurately by the PowerGlove (Fig 3). Higher differences found (in fast tasks in all subjects and in flexion task in subject 1) could be explained by the relative marker movement during the finger movements, having the largest effect on the segment orientation (Fig 4).

By not having to use a complete lab-based camera set-up with all its limitations like occlusion of markers, the PowerGlove is a flexible 3D measurement system that may facilitate the measurement of finger-interdependency in elderly or the measurement of hand motor symptoms in clinical practice very well. In particular in PD patients, the evaluation of tasks like finger tapping are part of the neurological exam in which for example the thumb/index amplitude and the MCP joint angles are important [5], whereas finger inter-dependency can be studied using finger flexion tasks with a focus on all three finger joints [4]. Objective measurements can be easily performed at home, in the hospital or even in the operating room.

For interpretation of the data of the PowerGlove in assessment of hand motor function the results as reported in this paper need to be taken into account. Furthermore, one needs to be aware of the optimal condition of application of the PowerGlove, such as a correct inclination angle of the hand for orientation estimated from the acceleration, or change in movement to optimally use the gyroscopes [12].

### Limitations of the study

The current study concerned a technical assessment of the performance of the PowerGlove in comparison to the conventional opto-electronic marker system. Therefore, in our opinion the three subjects included were sufficient to draw conclusions, since inter-subject differences are of less importance. Even, the evaluation could have been performed in only one subject such as has been done in other finger kinematic studies comparing systems or methods [12,15,16]. In addition, the variety of tasks show a wide range of applications of the PowerGlove. However, the multiple subjects show that performance of anatomical calibration, an individual procedure, might affect the accuracy of the results.

In this study we were not able to analyse the movement of the middle finger due to marker occlusion problems. During the measurement, three cameras were used to track the markers, however due to the hand position and the index finger being in the way, the middle finger could not be tracked very well. Using more cameras or a different marker set-up with e.g. small tripods with markers on the finger segments might have overcome the occlusion problem, although the latter could have impeded the finger movements. It could be expected though that measurement of the middle finger movement with the PowerGlove is comparable to the index finger, since a similar set-up and calibration of inertial sensor units is applied.

In literature, the CyberGlove (piezo-resistive technology) [11,24], WU-glove [25] and NeuroAssess Glove [26] (resistive bend sensors) are considered as accurate instrumented glove systems currently available, although calibration and fitting do influence the accuracy [11]. A direct comparison between such systems and the PowerGlove for the specific movement tasks included in this study could not been made, since these systems were currently not available for the authors and results on measurement of 3D angles per each individual joint were hard to find in literature. Previously, repeatability outcomes for total joint angles (i.e. sum of individual joint angles) in flat and flexed hand position have been reported [12], showing a similar or better repeatability for the PowerGlove.

## Conclusion

Median differences in finger joint angles between the inertial sensor units (PowerGlove) and an opto-electronic marker system ranged between 3 and 8deg. Smallest differences were observed in the flexion tasks. Larger differences observed in fast tasks such as tapping and circular pointing tasks were mainly differences in measured range of motion, due to skin movement artefacts caused by relative marker movements of the opto-electronic marker system, high velocity of the movement which exceeded the range of the inertial sensors or differences in segment calibrations between systems. For fast finger tapping, the thumb/index amplitude showed a median difference of 15.8mm.

The PowerGlove is a system that can be of value for measurement of various finger movement tasks in a variety of conditions and population in an ambulatory setting. For interpretation of the data of the PowerGlove in assessment of hand motor function the results as reported in this paper need to be taken into account.

## Supporting Information

S1 File Outcomes joint angles and positions per subject. (XLSX)

S2 File Markermovent per subject. (XLSX)

S3 File Angular velocity per subject. (XLSX)

## References

[1] Cole KJ, Cook KM, Hynes SM, Darling WG. Slowing of dexterous manipulation in old age: force and kinematic findings from the 'nut-and-rod' task. Exp Brain Res. 2010;201: 239–247. 10.1007/s00221-009-2030-z 19795110

[2] Latash ML, Shim JK, Shinohara M, Zatsiorsky VM. Changes in finger coordination and hand function with advanced age In: Motor control and learning. 2006 pp. 141–159.

[3] Lang CE, Schieber MH. Human finger independence: Limitations due to passive mechanical coupling versus active neuromuscular control. Journal of Neurophysiology. 2004;92: 2802–2810. 10.1152/jn.00480.2004 15212429

[4] van Beek N, van den Noort JC, Veltink PH, Selles R, Veeger HE, Maas H et al. Mechanical constraints of finger independence: linking tendon displacement with joint movement. In Proceedings of 25th congress of the International Society of Biomechanics. 2015;

[5] Fahn S, Elton RL. The UPDRS development Committee, 'Unified Parkinson's Disease Rating Scale' In: Fahn S, Marsden CD, Calne DB, Goldstein M, editors. Recent Developments in Parkinson's Disease. Florham Park, NJ: Macmillan Healthcare Information 1987 pp. 153–163.

[6] Zyluk A, Skala K. Hand disorders in the course of systemic and chronic diseases: a review. Pol Orthop Traumatol. 2014;79: 30–36. 24694790

[7] Parker WL. Evidence-based medicine: thumb carpometacarpal arthroplasty. Plast Reconstr Surg. 2013;132: 1706–1719. 10.1097/PRS.0b013e3182a807af 24281596

[8] Sultana SS, MacDermid JC, Grewal R, Rath S. The effectiveness of early mobilization after tendon transfers in the hand: a systematic review. J Hand Ther. 2013;26: 1–20. 10.1016/j.jht.2012.06.006 23116645

[9] Patrick SK, Denington AA, Gauthier MJA, Gillard DM, Prochazka A. Quantification of the UPDRS rigidity scale. Ieee Transactions on Neural Systems and Rehabilitation Engineering. 2001;9: 31–41. 10.1109/7333.918274 11482361

[10] Post B, Merkus MP, De Bie RMA, De Haan RJ, Speelman JD. Unified Parkinson's disease rating scale motor examination: Are ratings of nurses, residents in neurology, and movement disorders specialists interchangeable? Movement Disorders. 2005;20: 1577–1584. 10.1002/mds.20640 16116612

[11] Dipietro L, Sabatini AM, Dario P. A survey of glove-based systems and their applications. Ieee Transactions on Systems Man and Cybernetics Part C-Applications and Reviews. 2008;38: 461–482.

[12] Kortier HG, Sluiter VI, Roetenberg D, Veltink PH. Assessment of hand kinematics using inertial and magnetic sensors. Journal of Neuroengineering and Rehabilitation. 2014;11: 70–83. 10.1186/1743-0003-11-70 24746123 PMC4019393

[13] Nataraj R, Li ZM. Robust identification of three-dimensional thumb and index finger kinematics with a minimal set of markers. J Biomech Eng. 2013;135: 91002 10.1115/1.4024753 23775305 PMC3708711

[14] Shen ZL, Mondello TA, Nataraj R, Domalain MF, Li ZM. A digit alignment device for kinematic analysis of the thumb and index finger. Gait Posture. 2012;36: 643–645. 10.1016/j.gaitpost.2012.04.012 22633016 PMC3597988

[15] Goislard de Monsabert B, Visser JM, Vigouroux L, Van der Helm FC, Veeger HE. Comparison of three local frame definitions for the kinematic analysis of the fingers and the wrist. J Biomech. 2014;47: 2590–2597. 10.1016/j.jbiomech.2014.05.025 24998990

[16] Coupier J, Moiseev F, Feipel V, Rooze M, Van Sint JS. Motion representation of the long fingers: a proposal for the definitions of new anatomical frames. J Biomech. 2014;47: 1299–1306. 10.1016/j.jbiomech.2014.02.017 24612716

[17] Zatsiorsky VM, Li ZM, Latash ML. Enslaving effects in multi-finger force production. Exp Brain Res. 2000;131: 187–195. 10766271 10.1007/s002219900261

[18] Ryu JH, Miyata N, Kouchi M, Mochimaru M, Lee KH. Analysis of skin movement with respect to flexional bone motion using MR images of a hand. J Biomech. 2006;39: 844–852. 10.1016/j.jbiomech.2005.02.001 16488223

[19] Soderkvist I, Wedin PA. Determining the movements of the skeleton using well-configured markers. J Biomech. 1993;26: 1473–1477. 8308052 10.1016/0021-9290(93)90098-y

[20] de Vries WH, Veeger HE, Cutti AG, Baten C, Van der Helm FC. Functionally interpretable local coordinate systems for the upper extremity using inertial & magnetic measurement systems. J Biomech. 2010;43: 1983–1988. 10.1016/j.jbiomech.2010.03.007 20382385

[21] Iaquinto JM, Tsai R, Haynor DR, Fassbind MJ, Sangeorzan BJ, Ledoux WR. Marker-based validation of a biplane fluoroscopy system for quantifying foot kinematics. Med Eng Phys. 2014;36: 391–396. 10.1016/j.medengphy.2013.08.013 24075068

[22] van den Noort JC, Ferrari A, Cutti AG, Becher JG, Harlaar J. Gait analysis in children with cerebral palsy via inertial and magnetic sensors. Med Biol Eng Comput. 2013;51: 377–386. 10.1007/s11517-012-1006-5 23224902

[23] Cutti AG, Giovanardi A, Rocchi L, Davalli A, Sacchetti R. Ambulatory measurement of shoulder and elbow kinematics through inertial and magnetic sensors. Med Biol Eng Comput. 2008;46: 169–178. 10.1007/s11517-007-0296-5 18087742

[24] Kessler GD, Hodges LF, Walker N. Evaluation of the CyberGlove as a whole hand input device. ACM Trans Comput -Human Interaction. 1995;2: 263–283.

[25] Gentner R, Classen J. Development and evaluation of a low-cost sensor glove for assessment of human finger movements in neurophysiological settings. J Neurosci Methods. 2009;178: 138–147. doi: S0165-0270(08)00655-9 [pii]; 10.1016/j.jneumeth.2008.11.005 19056422

[26] Oess NP, Wanek J, Curt A. Design and evaluation of a low-cost instrumented glove for hand function assessment. J Neuroeng Rehabil. 2012;9: 2. doi: 1743-0003-9-2 [pii]; 10.1186/1743-0003-9-2 22248160 PMC3305482

