# Correction: Measuring 3D Hand and Finger Kinematics—A Comparison between Inertial Sensing and an Opto-Electronic Marker System

**DOI:** 10.1371/journal.pone.0193329

**Published:** 2018-02-16

**Authors:** Josien C. van den Noort, Henk G. Kortier, Nathalie van Beek, DirkJan H. E. J. Veeger, Peter H. Veltink

In the citation, the third author’s name is incorrect. The correct name is: van Beek N. The correction citation is: van den Noort JC, Kortier HG, van Beek N, Veeger DHEJ, Veltink PH (2016) Measuring 3D Hand and Finger Kinematics—A Comparison between Inertial Sensing and an Opto-Electronic Marker System. PLoS ONE 11(11): e0164889. https://doi.org/10.1371/journal.pone.0164889
